# The independent effects of age and sex in performance fatigability profile after a ramp incremental cycling test

**DOI:** 10.1007/s00421-025-05823-0

**Published:** 2025-06-04

**Authors:** Rafael A. Azevedo, Guillaume Y. Millet, Juan M. Murias

**Affiliations:** 1 Faculty of Kinesiology, University of Calgary, Calgary, Canada; 2 Applied Physiology and Nutrition Research Group, Center of Lifestyle Medicine, Faculdade de Medicina FMUSP, Universidade de São Paulo, São Paulo, Brazil; 3 Université Jean Monnet Saint-Etienne, Lyon 1, Université Savoie Mont-Blanc, Laboratoire Interuniversitaire de Biologie de la Motricité, F-42023 Saint-Etienne, France; 4 Institut Universitaire de France (IUF), Paris, France; 5 College of Health and Life Sciences, Hamad Bin Khalifa University, Doha, Qatar

**Keywords:** Neuromuscular function, Peripheral fatigue, Central fatigue, Aging

## Abstract

**Purpose:**

To investigate the effects of age and sex in performance fatigability profile after a ramp incremental (RI) test.

**Methods:**

Older females (*n* = 13; 66 ± 5 yrs) and males (*n* = 13; 68 ± 4 yrs), and young females (*n* = 11; 25 ± 5 yrs) and males (*n* = 12; 25 ± 4 yrs) performed a RI test immediately preceded and followed by performance fatigability assessments that included: knee-extension isometric maximal voluntary contraction (IMVC) and femoral nerve electrical stimuli during and after the IMVC to calculate voluntary activation (VA) and contractile function (e.g., potentiated doublets at 10 and 100 Hz, and single twitches). Maximal oxygen uptake (V̇O_2_max) and peak power output (POpeak) were measured.

**Results:**

Young females and males showed greater V̇O_2_max and POpeak compared to older counterparts (all *p* < 0.05). The IMVC declined more in young (females:  −27 ± 14%; males:  −44 ± 7%) than older (females:  −23 ± 9%; males:  −26 ± 9%) (*p* < 0.01), and in males compared to females (*p* < 0.01). Single twitch declined more in young (females:  −43 ± 15%; males:  −54 ± 15%) than older participants (females:  −33 ± 10%; males:  −27 ± 18%) (*p* = 0.01), without sex differences (*p* = 0.59). Similar responses were observed for 100 Hz and 10 Hz stimulus for age and sex (all *p* > 0.05). Voluntary activation was not different (*p* = 0.11) between young (females:  −5 ± 5%; males:  −8 ± 6%) and older (females:  −7 ± 6%; males:  −12 ± 6%), but declined less in females than males (*p* = 0.03). There was no age × sex interaction for any performance fatigability outcome (all *p* ≥ 0.06).

**Conclusion:**

Contractile function was more impaired in young than older participants, whereas males showed greater decline in VA than females. There was no combined effect of age and sex in performance fatigability responses.

## Introduction

Aging is commonly linked to decreases in performance of daily living activities and functional capacity (Paterson et al. 2004). Importantly, depending on the task characteristics (Weavil et al. 2018), the lower functional capacity in the elderly may lessen the development of exercise-induced fatigue compared to their younger counterparts. Debate remains about the potential mechanisms responsible for the diminished exercise-induced fatigue development in older (i.e., ≥ 60 years old) compared to young adults (i.e., 18–35 years old) as aging can negatively affect the capacity of the cardiopulmonary system (Roman et al. 2016) and muscular metabolism function (Terjung et al. 2002), which are interconnected to the development of fatigue symptoms (Thomas et al. 2018).

Exercise performance depends on the interaction between perceived fatigability (i.e., changes in perceptual responses during exercise) and performance fatigability (i.e., a decline in maximal muscle force/power production) (Enoka and Duchateau 2016). Traditionally, the profile of exercise-induced performance fatigability responses is characterized by reductions in voluntary activation and/or contractile function (Millet et al. 2011), wherein the responses of both components are dependent on the characteristics of the task, such type of muscle contraction and intensity, and population investigated (Hunter 2016; Thomas et al. 2018). For example, there is less decline in contractile function in older compared to young adults after constant load cycling exercise at an intensity corresponding to 80% of the ramp incremental (RI) peak power output (Weavil et al. 2018). Specifically, the diminished decline in contractile function in older compared to younger adults might be due to lower exercise-induced metabolic disturbance (Hunter et al. 1999) since aging is associated with less of a skeletal muscle glycolytic activity (Terjung et al. 2002) and diminished overall high-intensity exercise tolerance, probably as a result of lower cardiopulmonary capacity (Murias et al. 2010). Interestingly, the age effect on the decline of contractile function, when utilizing dynamic muscle contractions, seems to be intensity-dependent as the difference between young and older individuals decreases when the cycling bout is performed at power output (PO) supra-RI test (i.e., 30-s Wingate test) (Krüger et al. 2018, 2020). In fact, previous evidence indicates even a greater decline in contractile function in older compared to young individuals when the exercise (i.e., isotonic knee-extension at ﻿20% of maximal isometric voluntary force) was performed at maximum velocity/power (i.e., 4 min test duration at ~ 16% duty cycle) (Sundberg et al. 2018b, a). The mechanisms underpinning this response are still under investigation, but it has been suggested that aging might compromise metabolic economy when performing dynamic contractions at relatively high intensity (Fitzgerald et al. 2021), probably due to lower predominance of type II fibers to sustain high dynamic muscle contraction PO production (Gries et al. 2018). This is a relevant point because, as previously mentioned, the profile of performance fatigability is task- and population-dependent, and to date, there is no study that has investigated this profile after a RI test, which requires maximal aerobic PO production, achieves maximal aerobic capacity, and metabolic disturbance. Specifically, the RI is frequently utilized as the gold standard to assess cardiorespiratory fitness and overall health status (Laukkanen et al. 2016), and, as abovementioned, it possesses relevant task characteristics when comparing performance fatigability profile in old and young individuals.

Another relevant aspect regarding the aging process and the performance fatigability that require further investigation is the potential for sex-related differences when evaluating older compared to young adults (Solianik et al. 2017). For instance, young females show less decline in contractile function compared to males after cycling bouts at submaximal aerobic capacity (Ansdell et al. 2019, 2020). This is probably because, in general, females rely more on oxidative phosphorylation and have lower glycolytic activity capacity than males (Montero et al. 2018), which ultimately affects the performance fatigability profile after an exercise bout near or at the maximal aerobic capacity (Dominelli et al. 2017; Thomas et al. 2018). Importantly, even though some evidence suggested that sex differences in performance fatigability, cardiopulmonary, and metabolic responses to exercise are maintained throughout the lifespan (Gries et al. 2018; Sundberg et al. 2018b), the majority of the data on age-related effects are based on male participants and/or did not investigate potential sex differences as shown by a previous literature review on the topic (Krüger et al. 2018). Thus, further studies are needed to evaluate whether performance fatigability responses along with cardiopulmonary and metabolic responses following a RI test are affected differently in older compared to young female and male participants.

Therefore, the aims of the present study were to investigate whether the profile of performance fatigability (i.e., reductions in voluntary activation and contractile function variables) after a RI test to task failure was affected by an interaction effect of the age and the sex of the participants. Our hypotheses were that the profile of performance fatigability would be characterized by lower decline in maximal voluntary force and contractile function in older compared to young adults, and in females compared to males after a RI test to task failure, thus maintaining in the older adults the sex-related differences previously reported for the young adults and not showing a combination of age and sex effects.

## Methods

*Participants.* The sample was composed of older females (*n* = 13; age range, 60–74 years old) and males (*n* = 13; age range, 62–74 years old), and young females (*n* = 11; age range, 20–33 years old) and males (*n* = 12; age range, 18–31 years old) (*see results section for participants’ characteristics*). The sample size calculation was performed in G*Power software (version 3.1.9.2), and it was based on the effect size from a previous study which evaluated the effects of high-intensity cycling in isometric maximal voluntary contractions (IMVC) of knee extensors between older and young individuals (Krüger et al. 2020). For an expected effect size of 0.86 for f tests (or Cohen’s d of 1.72), power of 0.90 and alpha level of 0.05, a total sample size of 20 participants (i.e., 5 participants per group) was estimated. Nonetheless, we collected at least 11 participants per group because performance fatigability measurements display greater variability in older compared to young participants (Hunter et al. 2016). None of the participants were undergoing any medical treatment that could potentially alter their cardiopulmonary, neuromuscular, and metabolic responses to maximal exercise. The age limit for young and older participants was set at between 18–35 and 60–75 years old, respectively. This was based on previous research that indicated that aerobic capacity declined after the age of 35 years (Hunter and Stevens 2013), and muscle force production capacity had a sharp decline beyond 75 years of age (Sundberg et al. 2018b). All participants were cleared to perform maximal exercise after an initial screening including: (i) having a physical activity level being above 24 points obtained from the “Godin–Shephard Leisure-Time Physical Activity Questionnaire” (Amireault and Godin 2015); and (ii) passing a “Physical Activity Readiness Questionnaire” (PAR-Q). Additionally, all older participants had continuous electrocardiogram recordings at rest and during exercise to minimize the risk of not detecting any potential abnormal cardiac responses in this population. Young female participants self-reported a menstrual cycle length of 28 ± 5 days, and four participants were taking hormonal contraceptives. All participant signed an informed consent form. All procedures were approved by the Conjoint Health Research Ethics Board at the University of Calgary (REB18-0916). The dataset utilized in this study was designed to answer a variety of different research questions, which included performance fatigability assessments in young population only and at constant load cycling bouts of exercise. Thus, some of the data from young individuals have been previously presented as descriptive values, such as maximal oxygen uptake (V̇O_2_max) and peak PO (POpeak) from the RI test (Azevedo et al. 2021). Importantly, the order of the sessions in the current and the previous study followed the same pattern (i.e., the RI test was the first exercise protocol for all participants).

**Fig. 1:**
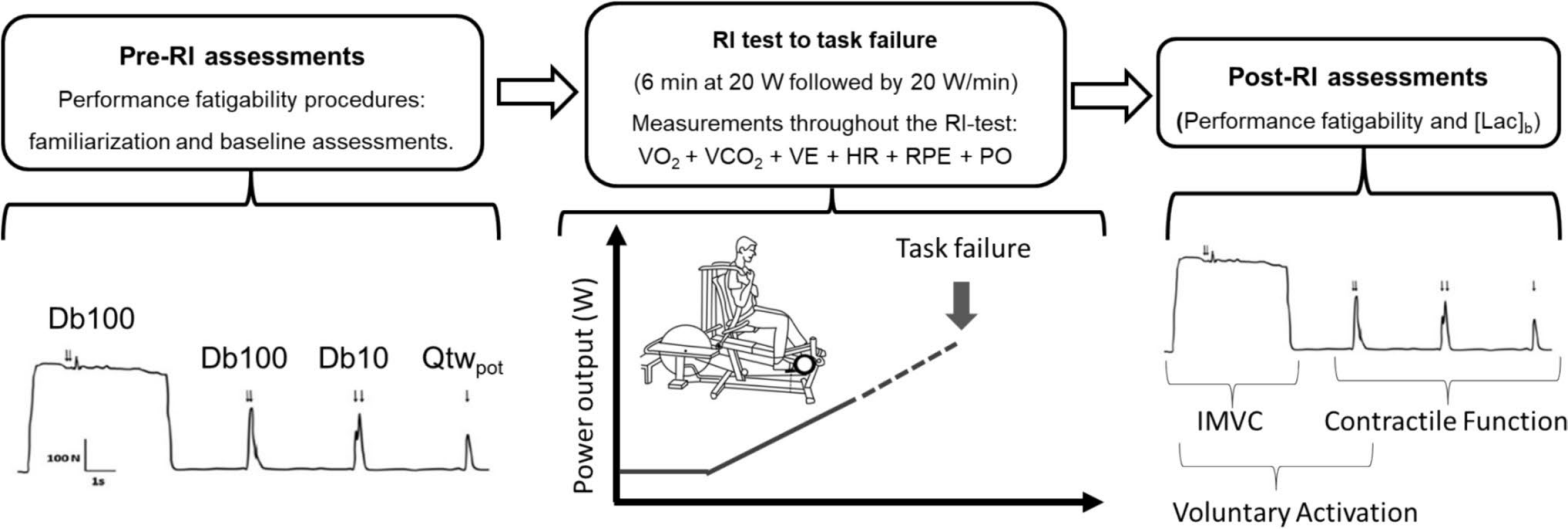
Schematic representation of the study design. *RI* Ramp incremental test, *IMVC* Isometric maximal voluntary contraction, *VA* Voluntary activation, *Db100* 100 Hz paired pulses evoked torque, *Db10* 10 Hz paired pulses evoked torque, Qtw_pot_ Single-pulse-evoked torque, *V̇O*_2_ Oxygen consumption, *V̇CO*_*2*_ Carbon dioxide production, *V̇E* Minute ventilation, *HR* Heart rate, *PO* Power output, *RPE* Rating of perceived exertion, *[Lac]*_*b*_ Blood lactate concentration

*Experimental protocol.* Figure 1 depicts the overall experimental design. Before any physical testing took place, stature, body mass, and body composition (i.e., % body fat and lean tissue mass using dual energy X-ray absorptiometry (Discovery QDR Series, Hologic, Inc., MA) were assessed. The exercise testing session consisted of 6 min baseline cycling at 20 W followed by a RI test with power output increments of 20 W·min^−1^ to task failure, where pulmonary gas exchange, ventilation, and heart rate responses were continuously recorded. Performance fatigability was assessed before and immediately after the RI test, by femoral nerve electrical stimuli evoked during and after IMVC of knee extensors. Rating of perceived exertion and blood lactate concentration were assessed post-RI test performance fatigability measurements. All tests were conducted in an environmentally controlled room (temperature: 18–21 °C; humidity 50–60%). Prior to the session, all participants were instructed to avoid consumption of food and caffeinated beverages for at least 2 h and 8 h, respectively, and to abstain from vigorous physical activity for 24 h.

### Data collection

*Cycling exercise.* Cycling was performed on an innovative and custom-built electromagnetically braked cycle ergometer (Velotron, RacerMate, Seattle, WA) (Doyle-Baker et al. 2018). Importantly, this ergometer allowed performance fatigability assessments immediately after exercise cessation. Thus, any recovery effect was minimized to better represent the fatigue effect in contractile function and/or voluntary activation responses to exercise (Doyle-Baker et al. 2018; Krüger et al. 2019). During the RI test, participants adopted their preferred cadence and task failure was determined when participants could no longer maintain a cycling cadence of at least 60 rpm for more than 5 s, or at volitional exhaustion despite strong verbal encouragement. Participants were blinded to the work rate and elapsed time but received visual feedback on their cadence.

*Gas exchange, ventilator, and heart rate variables*. All gas exchange and ventilatory responses were measured breath by breath with a metabolic cart (Quark, Cosmed, Rome, Italy). The system consisted of a low dead space turbine as well as O_2_ and carbon dioxide (CO_2_) gas analyzers. These were calibrated with a syringe of known volume (3 L) and a gas mixture of known concentration (16% O_2_; 5% CO_2_; balance N2), respectively. Heart rate (HR) was measured by a heart rate monitor (Garmin International, Schaffhausen, Switzerland) connected to the metabolic cart.

*Performance fatigability assessments.* Voluntary and electrically evoked torques were recorded using a wireless PowerForce pedal force analysis system (Model PF 1.0.0, Radlabor GmbH, Freiburg, Germany) located between the pedal and the crank that had been previously validated (Doyle-Baker et al. 2018), which allowed virtually no time delay between exercise cessation and performance fatigability assessments (26). Briefly, for the performance fatigability assessments, the pedals were locked parallel to the ground, which allowed fixing the hip angle at 100° while the knee and the ankle angles were fixed at 90°. Additionally, the participants were secured by noncompliant straps at the hip and the chest to minimize any interference on knee-extension force. The amplitude of force measurements ranged from − 500 to + 1500 N and the accuracy error for the system is < 2.35%. The torque produced by the knee extensors was sampled at 500 Hz and converted from analog to digital using Imago Record (version 8.50, Radlabor GmbH) software. Visual feedback on torque was provided in real time on a computer screen by converting the PowerForce signal to analog signal and exported via a data acquisition card (NI PCI-6229, National Instruments, Austin, TX) and connector block (BNC-2111, National Instruments) to a PowerLab system (16/35, ADInstruments, Bella Vista, Australia). The data recorded using Imago Record were converted to a text file and analyzed offline using the Labchart 8 software (ADInstruments).

Femoral nerve transcutaneous electrically evoked contractions of the dominant-leg knee extensor muscles were induced using a high-voltage stimulator (DS71, Digitimer, Welwyn Garden City, Hertfordshire, UK) to deliver a monophasic rectangular pulse (1-ms duration). The femoral nerve was stimulated via a monopolar 10-mm diameter cathode electrode (Meditrace 100, Covidien) placed on the inguinal triangle and 50 × 90 mm rectangular anode electrode (Durastick Plus, DJO Global, Vista, CA) in the gluteal fold. For the optimum stimulation intensity, single stimuli were delivered incrementally (starting at 10 mA and increment of 10 mA every 30 s) until a plateau in twitch torque and maximal M wave amplitudes were reached (i.e., peak-to-peak amplitude in response to single stimuli measured as the absolute difference between maximum and minimum points of the biphasic M wave in mV). Supramaximal stimulation at 130% of the intensity to elicit maximal twitch and M wave amplitudes was delivered to confirm supramaximal intensity (older females, 112 ± 29 mA; older males, 165 ± 35 mA; young females, 74 ± 17 mA; young males, 70 ± 17 mA).

After establishing the optimal electrical nerve stimulation intensity, all participants underwent thorough familiarizations with performance fatigability procedures. Briefly, the participants performed a standardized warm-up consisting of five 5 s contractions of the knee extensors, interspersed by 30-s rest periods, at intensities corresponding to 50, 60, 70, and 80% of the maximum subjective torque, followed by one IMVC. Thereafter, participants performed three 5 s IMVCs, interspersed by 60 s rest period. Visual torque feedback and verbal encouragement were provided during all IMVCs. Finally, participants underwent the electrical nerve stimulation protocol which consisted of a 5 s IMVC and high-frequency (100 Hz, Db100), low-frequency (10 Hz, Db10) paired stimuli and a single potentiated stimulus (Qtw_pot_). During the 5 s IMVC, a superimposed high-frequency (i.e., 100 Hz) twitch was applied when the participants produced maximal torque and once the muscle was relaxed, electrically evoked torques were elicited by Db100, Db10 and Qtw_pot_ interspaced by 2 s.

*Electromyography.* Electromyographic activity of the dominant-leg knee extensor VL muscle was recorded via self-adhesive Ag–AgCl surface electrodes (Meditrace 100, Covidien, Mansfield, MA) in a bipolar configuration with a 30 mm interelectrode distance and the reference on the patella. To prevent movement artifact, the electrode wires were taped to the skin using adhesive tape. A low impedance (< 5 kΩ) between electrodes was obtained by shaving and gently abrading the skin and then cleaning it with isopropyl alcohol. EMG signals were converted from analog to digital at a sampling rate of 2000 Hz by PowerLab system (16/35, ADInstruments) and octal bio-amplifier (ML 138, ADInstruments; common mode rejection ratio = 85 dB, gain = 500; low-pass Bessel filter of fourth-order and high-pass filter of first-order) with a bandpass width (5–500 Hz) and analyzed offline using the Labchart 8 software (ADInstruments).

*Rating of perceived exertion and blood lactate concentration.* The rating of perceived exertion (RPE) was assessed using a 15-points category scale (6–20 Borg) (Borg 1982). The instructions for RPE scale were standardized (Azevedo et al. 2023a). Peak blood lactate concentration ([Lac]_b_) measurement was completed immediately after the performance fatigability assessments, by wiping a finger with an alcohol swab, followed by a finger prick, and collection of a 20 μl blood sample with a capillary tube, which was mixed in an EKF pre-filled safe lock plastic tube for analysis (EKF Biosen C-Line Analyzer, Barleben, Germany).

### Data analyses

V̇O_2_ data during the RI tests were cleaned by removing data points laying ± 3 standard deviation (SD) from the local mean and linearly interpolating the data to 1-s intervals (Origin, Origin Lab, Northampton, MA). V̇O_2_max, maximal minute ventilation (V̇_E_max) and respiratory exchange ratio (RERmax, i.e., V̇CO_2_/V̇O_2_) were computed as the highest value from a 20 s rolling average during the RI test (Murias et al. 2018; Iannetta et al. 2020). The POpeak corresponded to the highest PO value recorded at the end of the RI test for each participant. The maximal HR (HRmax) was obtained as the highest value achieved during the RI test. Previous findings support that aerobic maximal metabolic rate (i.e., V̇O_2_max) can be achieved during a RI test when participants are pushed to perform maximal efforts independently of the slope of the ramp (Murias et al. 2018). To add confidence to the achievement of a maximal effort, the following criteria were also utilized (Howley et al. 1995): (i) subjective evidence of exhaustion (RPE ≥ 17), (ii) HRmax ≥ 90% age predicted maximum (Tanaka et al. 2001), and (iii) RERmax ≥ 1.10.

The IMVC was the marker of global performance fatigability and the voluntary activation (VA) and the evoked torques on relaxed muscle, assumed to be the voluntary activation (i.e., central) and the contractile function (i.e., peripheral) components of performance fatigability. The profile of performance fatigability was characterized as the changes in percentage values from baseline to immediately after the RI task failure for IMVC, VA and contractile function variables. Maximal voluntary torque was calculated as the highest IMVC prior to the cycling exercise, and electrically evoked torque from the doublets and single pulse was determined as the peak torque of each stimulation on the relaxed muscle. The contractile function changes were characterized by calculating the pre- to post-RI test changes in Db100, the ratio between Db10 and Db100 (Db10:100), Q_twpot_, and the M wave amplitude in mV for the VL muscle. The VA changes were characterized by the pre- to post-RI test assessed by a superimposed paired-pulse technique as previously described by Strojnik and Komi (Strojnik and Komi 1998):

VA (%) = 100 – D × (IMVC_Db100_/ IMVC_peak_) / Db100 × 100.

where IMVC_Db100_ is the voluntary torque when superimposed Db100 was delivered, IMVC_peak_ is the highest torque during the IMVC before the superimposed Db100, D is the difference between the torque level at the time of IMVC_Db100_ and the maximum torque during superimposed Db100, and Db100 is the electrically evoked torque on relaxed muscle two seconds after IMVC. Recommendations related to instructions, practice, visual feedback of performance, and standardized verbal encouragement were adopted in the present study to ensure the maximum effort of participants during the IMVCs and performance fatigability parameters assessments (Gandevia 2001).

### Statistical analysis

Normal data distribution was confirmed by Shapiro and Wilk test, and the results are reported as mean ± one SD. Comparisons among older and young females and males were performed by a two-way ANOVA composed by age and sex as main factors. When F values were significant for main effects, Bonferroni post hoc tests were used to determine where differences existed. The effect sizes for ANOVA comparisons were computed as partial eta-squared (${\eta }_{p}^{2}$) and evaluated as small (< 0.02), medium (0.02–0.26), or large (> 0.26) (Bakeman 2005). The significance level was set at *P* ≤ 0.05. All statistical analyses were performed using a statistical software package (Statistica, version 10.0, Tulsa, OK).

## Results

### Participants’ characteristics, neuromuscular function, and RI test results

Tables 1, 2, and 3 show the comparisons, with their respective p values and effect sizes (i.e., ${\eta }_{p}^{2}$), between the older and the young females, and male participants for sample characteristics, neuromuscular function, and RI results, respectively. Overall, and by design, there was a significant difference in age between groups, but not between sexes (Table 1). There was no difference between groups for physical activity level as acquired by Godin’s questionnaire (Amireault and Godin 2015).

**Table 1:** Sample characteristics for older and young females and males

	OLDER		YOUNG		*p* values and effect sizes		
	♀ (n = 13)	♂ (n = 13)	♀ (n = 11)	♂ (n = 12)	Age effect	Sex effect	Age × sex interaction
Age (yrs)	66 ± 5	68 ± 4	25 ± 5^#^	25 ± 4^#^	**p < 0.001** ${\eta }_{p}^{2}$ = 0.95	p = 0.60 ${\eta }_{p}^{2}$ < 0.01	p = 0.54 ${\eta }_{p}^{2}$ < 0.01
Body mass (kg)	59 ± 12	77 ± 8^⚥^	59 ± 6	75 ± 11^⚥^	p = 0.74 ${\eta }_{p}^{2}$ < 0.01	**p < 0.01** ${\eta }_{p}^{2}$ = 0.45	p = 0.62 ${\eta }_{p}^{2}$ < 0.01
Body stature (cm)	161 ± 7	174 ± 8^⚥^	169 ± 5^#^	179 ± 11^#,⚥^	**p < 0.01** ${\eta }_{p}^{2}$ = 0.17	**p < 0.01** ${\eta }_{p}^{2}$ = 0.44	p = 0.56 ${\eta }_{p}^{2}$ < 0.01
Body fat (%)	31 ± 8	25 ± 6^⚥^	18 ± 3^#^	13 ± 2^#,⚥^	**p < 001** ${\eta }_{p}^{2}$ = 0.58	**p < 0.01** ${\eta }_{p}^{2}$ = 0.24	p = 0.89 ${\eta }_{p}^{2}$ < 0.01
LBM (kg)	38 ± 4	55 ± 5^⚥^	48 ± 5^#^	64 ± 8^#,⚥^	**p < 0.01** ${\eta }_{p}^{2}$ = 0.43	**p < 0.01** ${\eta }_{p}^{2}$ = 0.69	p = 0.62 ${\eta }_{p}^{2}$ < 0.01
Physical Activity* Level (a.u.)	46 ± 17	48 ± 15	45 ± 22	43 ± 20	p = 0.37 ${\eta }_{p}^{2}$ = 0.01	p = 0.72 ${\eta }_{p}^{2}$ < 0.01	p = 0.70 ${\eta }_{p}^{2}$ < 0.01

^*^According to Godin’s questionnaire scores; Lean body mass (LBM); ♀, females; ♂, males. #, age differences; ⚥, sex differences

Bold indicates the statistical significant values

**Table 2:** Neuromuscular function at baseline for older and young females and males

	OLDER		YOUNG		p values		
	♀	♂	♀	♂	Age effect	Sex effect	Age × sex interaction
IMVC (N)	191 ± 20	356 ± 171^⚥^	246 ± 54^#^	468 ± 113^#,⚥^	**p = 0.01** ${\eta }_{p}^{2}$ = 0.13	**p < 0.01** ${\eta }_{p}^{2}$ = 0.46	p = 0.35 ${\eta }_{p}^{2}$ = 0.01
VA (%)	98 ± 3	96 ± 5	99 ± 2	96 ± 4	p = 0.73 ${\eta }_{p}^{2}$ < 0.01	p = 0.28 ${\eta }_{p}^{2}$ < 0.01	p = 0.71 ${\eta }_{p}^{2}$ < 0.01
Db100 (N)	113 ± 16	178 ± 40^⚥^	130 ± 19^#^	223 ± 35^#,⚥^	**p < 0.01** ${\eta }_{p}^{2}$ = 0.23	**p < 0.01** ${\eta }_{p}^{2}$ = 0.65	p = 0.09 ${\eta }_{p}^{2}$ = 0.06
Db10:100	1.03 ± 0.07	1.04 ± 0.09	0.97 ± 0.09	1.02 ± 0.06	p = 0.09 ${\eta }_{p}^{2}$ = 0.05	p = 0.19 ${\eta }_{p}^{2}$ = 0.03	p = 0.53 ${\eta }_{p}^{2}$ < 0.01
Qtw_pot_ (N)	81 ± 13	128 ± 34^⚥^	91 ± 14^#^	167 ± 33^#,⚥^	**p < 0.01** ${\eta }_{p}^{2}$ = 0.19	**p < 0.01** ${\eta }_{p}^{2}$ = 0.60	p = 0.06 ${\eta }_{p}^{2}$ = 0.07
M wave (mV)	12 ± 4	16 ± 5^⚥^	15 ± 5	17 ± 4^⚥^	p = 0.08 ${\eta }_{p}^{2}$ = 0.06	**p = 0.02** ${\eta }_{p}^{2}$ = 0.11	p = 0.43 ${\eta }_{p}^{2}$ = 0.01

*IMVC* Isometric maximal voluntary contraction, *VA* Voluntary activation, 100 Hz paired pulses evoked torque (Db100); Ratio between 10 and 100 Hz evoked torques (Db10:100); Single-pulse-evoked torque (Qtw_pot_); Peak-to-peak muscle compound amplitude (M wave). ♀, females; ♂, males. #, age differences; ⚥, sex differences

Bold indicates the statistical significant values

**Table 3:** Ramp incremental tests results at task failure for older and younger females and males

	OLDER		YOUNG		p values		
	♀	♂	♀	♂	Age effect	Sex effect	Age × sex interaction
V̇O_2_max (L·min^−1^)	2.04 ± 0.34	2.93 ± 0.50^⚥^	2.59 ± 0.42^#^	3.59 ± 0.33^#,⚥^	**p < 0.01** ${\eta }_{p}^{2}$ = 0.38	**p < 0.01** ${\eta }_{p}^{2}$ = 0.59	p = 0.57 ${\eta }_{p}^{2}$ < 0.01
V̇O_2_max (mL·kg^−1^·min^−1^)	36.2 ± 9.1	38.3 ± 7.4	44.1 ± 7.4^#^	49.1 ± 8.3^#^	**p < 0.01** ${\eta }_{p}^{2}$ = 0.26	p = 0.13 ${\eta }_{p}^{2}$ = 0.04	p = 0.53 ${\eta }_{p}^{2}$ < 0.01
POpeak (W)	159 ± 29	232 ± 44^⚥^	214 ± 44^#^	253 ± 23^#,⚥^	**p < 0.01** ${\eta }_{p}^{2}$ = 0.23	**p < 0.01** ${\eta }_{p}^{2}$ = 0.39	p = 0.09 ${\eta }_{p}^{2}$ = 0.05
POpeak (W·kg^−1^)	2.8 ± 0.8	3.0 ± 0.6	3.6 ± 0.8^#^	3.5 ± 0.7^#^	**p < 0.01** ${\eta }_{p}^{2}$ = 0.17	p = 0.91 ${\eta }_{p}^{2}$ < 0.01	p = 0.35 ${\eta }_{p}^{2}$ = 0.01
V̇_E_max (L·min^−1^)	102 ± 1	132 ± 28^⚥^	123 ± 23^#^	167 ± 25^#,⚥^	**p < 0.01** ${\eta }_{p}^{2}$ = 0.28	**p < 0.01** ${\eta }_{p}^{2}$ = 0.41	p = 0.30 ${\eta }_{p}^{2}$ = 0.02
HRmax (bpm)	164 ± 11	162 ± 11	184 ± 9^#^	185 ± 6^#^	**p < 0.01** ${\eta }_{p}^{2}$ = 0.58	p = 0.83 ${\eta }_{p}^{2}$ < 0.01	p = 0.51 ${\eta }_{p}^{2}$ < 0.01
Age-estimated HRmax (%)	105 ± 6	102 ± 6	97 ± 4	97 ± 3	p = 0.36 ${\eta }_{p}^{2}$ = 0.01	p = 0.32 ${\eta }_{p}^{2}$ = 0.02	p = 0.35 ${\eta }_{p}^{2}$ = 0.01
RERmax (a.u.)	1.17 ± 0.06	1.16 ± 0.08	1.17 ± 0.08	1.11 ± 0.06	p = 0.23 ${\eta }_{p}^{2}$ = 0.03	p = 0.10 ${\eta }_{p}^{2}$ = 0.05	p = 0.14 ${\eta }_{p}^{2}$ = 0.04
RPEmax (6–20)	18 ± 1	18 ± 1	19 ± 1	19 ± 1	p = 0.23 ${\eta }_{p}^{2}$ = 0.03	p = 0.10 ${\eta }_{p}^{2}$ = 0.05	p = 0.14 ${\eta }_{p}^{2}$ = 0.04
[Lac]_b_ (mM)	6.9 ± 1.2	6.4 ± 1.1	9.0 ± 1.6^#^	10.2 ± 1.8^#^	**p < 0.01** ${{\varvec{\eta}}}_{{\varvec{p}}}^{2}$ **= 0.54**	p = 0.37 ${\eta }_{p}^{2}$ = 0.01	**p = 0.03** ${{\varvec{\eta}}}_{{\varvec{p}}}^{2}$ **= 0.09**

*V̇O*_*2*_ Oxygen uptake, *PO* peak Peak power output , *V̇*_*E*_ Minute ventilation , *HR* Heart rate, *RER* Respiratory exchange ratio , *RPE* Rating of perceived exertion , *[Lac]*_*b*_ Post-RI blood lactate concentration , ♀, females, ♂, males.^#^, age difference, ⚥, sex differences

In relation to neuromuscular function at baseline (Table 2), whereas the values for IMVC and contractile function (i.e., D100 and Qtw_pot_) were lower in older compared to young participants, and in females compared to males, there was no difference in VA between age and sex groups. Additionally, Db10:100 was not different between age and sex groups, and M wave amplitude was not different between older and young participants, but it was lower in females compared to males.

Finally, for ramp incremental tests results (Table 3), V̇O_2_max (i.e., in mL·min^−1^) and POpeak (i.e., in W) were lower in older compared to younger participants and in females compared to males. However, relative to body mass, V̇O_2_max (mL·kg^−1^·min^−1^) and POmax (W·kg^−1^) were lower in older compared young participants, but similar between females and males regardless the age. The HRmax was lower in older compared to younger participants regardless the sex, but there were no age- and sex-related differences for HRmax normalized by the age-estimated maximum. Finally, [Lac]_b_ values were lower in older compared to younger adults, but no difference existed between sexes.

**Fig. 2:**
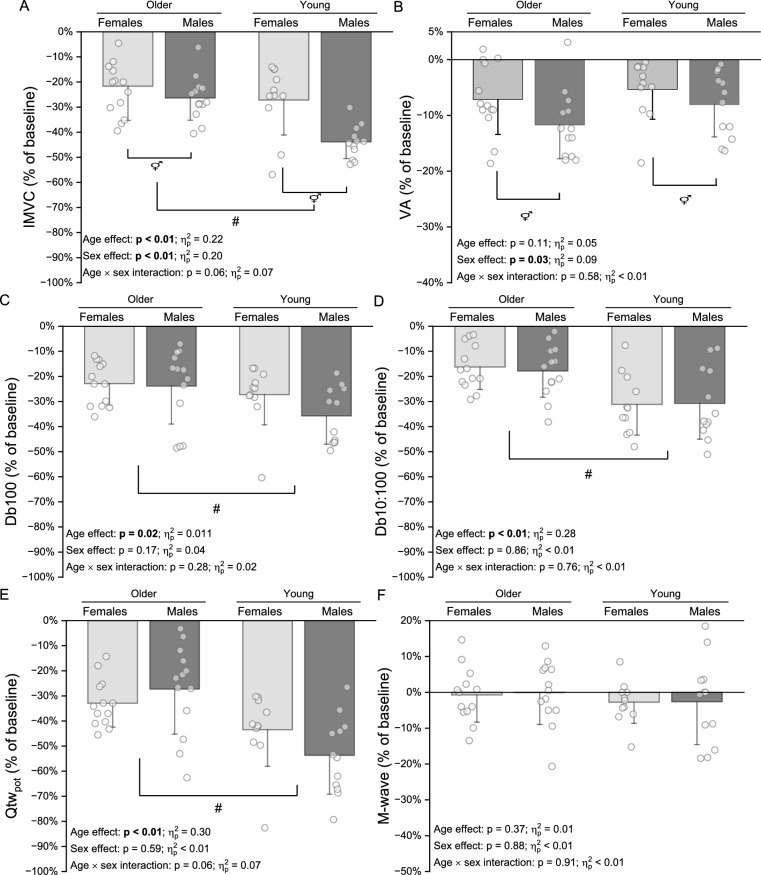
Performance fatigability responses in normalized values from baseline to immediately post-RI test in old and young females and males. Panel A, isometric maximal voluntary contraction (IMVC); Panel B, voluntary activation (VA); Panel C, 100 Hz paired pulses evoked torque (Db100); Panel D, ratio between 10 and 100 Hz evoked torques (Db10:100); Panel E, single-pulse-evoked torque (Qtw_pot_). #, age differences; ⚥, sex differences

*Performance fatigability responses.* Figure 2 shows the comparisons, with their respective p values and effect sizes (i.e., ${\eta }_{p}^{2}$), for performance fatigability variables in older and younger females and males after the RI test. There was a smaller decline in IMVC for older compared to younger participants and in females compared to males. There was no difference in VA between older and younger participants, but there was a smaller decline in females compared to males. For contractile function variables, there was smaller decline in older compared to young participants for Db100, Db10:100 and Qtw_pot_, with no sex differences observed for any of these variables. There was no difference for M wave between age and/or sex groups.

## Discussion

The novel findings of this study were that performance fatigability after the RI test was not affected by the combined effects of age and sex, but rather by their independent effects in contractile function and voluntary activation. Specifically, this study is the first to show that: (i) contractile function declined less in older compare to younger individuals regardless the sex, up to the maximal aerobic capacity and peak PO production, as assessed in the RI test; (ii) compared to males, females showed less decline in voluntary activation regardless the age; and (iii) there were lower metabolic rates (i.e., V̇O_2_max) and lactate accumulation in older compared to young participants regardless the sex, which might have been the underpinning mechanisms of performance fatigability changes, specifically those variables related to contractile function (i.e., Db100, Db10:100 and Qtw_pot_). Taken together, these data indicate that age and sex effects in performance fatigability are present after a RI test, but might differently affect its components as represented by contractile function and VA responses according to age and sex of the individual.

### Interaction effect of age and sex in performance fatigability after a ramp incremental test

A novel finding of the current study was that there was no interaction effect of age and sex in performance fatigability responses after a RI test (all age × sex interaction effects were *p* ≥ 0.06), which is in accordance with out initial hypothesis. Overall, the current results corroborate previous studies that showed an absence of interaction effect of age and sex in performance fatigability profile (Kent-Braun et al. 2002; Hunter et al. 2004; Sundberg et al. 2018b). However, it must be highlighted that, at least numerically, IMVC and Qtw_pot_ variables declined more in young males compared to the other groups (i.e., see Fig. 2A and E for details). In this context, there is a debate regarding an interaction effect of age and sex for performance fatigability after an exercise bout since some studies showed an interaction effect (Ditor and Hicks 2000; Solianik et al. 2017), but this is not a universal finding (Kent-Braun et al. 2002; Sundberg et al. 2018b). For example, the greatest decline in performance fatigability outcomes (i.e., decline in IMVC) has been shown in younger males followed by older males compared to younger and older females (Ditor and Hicks 2000; Solianik et al. 2017). Additionally, the changes in performance fatigability parameters (i.e., IMVC) seem to be of smaller amplitude between younger and older females compared to male counterpart responses after maximal isometric contractions of different durations (i.e., 120 s and 3 min intermittent protocol) (Ditor and Hicks 2000; Solianik et al. 2017). Based on this finding, it could be expected that the greatest decline in performance fatigability parameters would occur in younger and older males, but minimal changes would exist between younger and older females, ultimately eliciting an age-and-sex interaction effect, which was not the case in our findings.

The absence of an interaction effect of age and sex in the current study might be connected with the characteristics of the task herein utilized since the type of contraction, muscle mass involved and, specially, the exercise intensity may directly interfere in the performance fatigability profile (Hunter 2018; Thomas et al. 2018). For example, when exercise bouts are performed within moderate- and/or heavy-intensity domains, older individuals and younger females show lower exercise-induced metabolic disturbance compared to their younger male counterparts, respectively. This lower exercise-induced metabolic disturbance might be a result of the greater relative area of type I fibers (Gries et al. 2018), reliance on oxidative phosphorylation (Russ et al. 2005), and/or lower local ischemia during the concentric phase of dynamic contractions (Hunter et al. 2004) in older individuals and young females compared to their counterparts. On the other hand, when exercise intensity surpasses the upper boundary of the heavy domain, the rate of intramuscular metabolite accumulation is exacerbated, regardless of age and sex of the individual (Sundberg et al. 2017, 2018b; Grosicki et al. 2022), thus relying in similar mechanisms of performance fatigability development. Nonetheless, it must be highlighted that aging is indeed accompanied by lower absolute exercise-induced metabolic disturbance response (Kent-Braun et al. 2002; Kent-Braun 2009), and thus lower magnitude of performance fatigability changes, regardless of sex of the individual (Weavil et al. 2018; Ansdell et al. 2020). Therefore, it could be speculated that the lack of age-and-sex interaction in the current results might have been a result of the exercise intensity performed to the upper limit of aerobic capacity, which might have led to a common underpinning mechanism of performance fatigability development. Another relevant aspect to keep in mind when interpreting our results is that the older participants herein utilized were relatively fit compared to the overall population within the same age spectrum (Gries et al. 2018), and with similar physical activity level as shown in Table 1. Thus, it is currently uncertain with the fitness level which could mask any age-related effect in performance fatigability and more research is needed on this topic.

### Effects of age and sex in performance fatigability components after a ramp incremental test

The present study showed that performance fatigability, as represented by changes in IMVC, was affected by age and sex although without an interaction effect. Specifically, IMVC declined less in older compared to younger participants, and in females compared to males after the cycling RI test to task failure (Fig. 2). Interestingly, these IMVC responses were accompanied by distinct effects of age and sex in contractile function and VA components of performance fatigability. In relation to contractile function changes, our results showed that younger participants had a greater decline in contractile function compared to their older counterparts, regardless the sex. Indeed, previous findings have shown less decline in contractile function with aging when the cycling bout was performed either within the heavy (Krüger et al. 2020) or the severe (Weavil et al. 2018) domain, no age-related effect was found in contractile function parameters (i.e., Db100, Db10:100 and Qtw_pot_) after a 30-s all-out supra-RI POpeak cycling test was performed (i.e., Wingate test) (Krüger et al. 2020). Of note, it must be briefly highlighted that those contractile function parameters are meant to represent different mechanisms within the excitation–contraction cycle, whereas the Db100 responses supposedly represent the actin–myosin cycle, the Db10:100 may represent the exercise-induced disruption in Ca^2+^ handling within the sarcoplasmic reticulum and muscle fiber cytosol. It must be highlighted that Db100 and Db10:100 parameters are often utilized in clinical and/or general population (i.e., sedentary and unmotivated participants) to characterize the exercise-induced impairments in contractile function because of lower discomfort from electrical nerve stimulations compared to the gold standard technique (i.e., 1-s tetani trains) (Verges et al. 2009), but some limitations have been recently pointed out regarding the sensitivity of the Db10:100 to detect low-frequency fatigue (or prolonged low-frequency force depression, PLFFD), in particular the fact that Db100 does not evoke maximal Ca^2+^ release (Ruggiero et al. 2019). Finally, the Qtw_pot_ is the most common parameter to represent overall contractile function and to acquire the M wave responses (Millet et al. 2011). Thus, our study is the first to show that aging diminishes the decline in all contractile function parameters up to the maximal aerobic capacity (i.e., V̇O_2_max was achieved at RI task failure).

Regarding the absence of sex effect in contractile function, even though previous findings have suggested that contractile function has less of a decline in females compared to males (Ansdell et al. 2020; Azevedo et al. 2021), the present study and recent data (Azevedo et al. 2023a) do not support this assumption when the fatiguing task is a cycling RI test. It has been speculated that as exercise intensity increases, the greater reliance on O_2_ delivery to support an increase in V̇O_2_ within the working muscles that females typically exhibit compared to males is abolished due to high internal pressure and local ischemia (Russ et al. 2003; Billaut and Bishop 2012), which ultimately may lead to similar metabolic disturbance and contractile function responses between sexes (Billaut and Bishop 2012; Azevedo et al. 2023a). Indeed, there were similar peak responses of V̇O_2_max and [Lac]_b_ at RI task failure between sexes, but not between age groups (Table 3). This reinforces the speculation that the sex differences in contractile function might have been abolished because of the similar metabolic disturbances achieved.

In relation to VA responses, there was no effect of age, but females showed less decline compared to males. The current literature on the topic shows sparse and conflicting results as some studies have shown greater decline in VA after cycling exercise in young individuals (Weavil et al. 2018), whereas others have not (Fitzgerald et al. 2021). Nonetheless, to date, this is the first study to show that changes in VA after a RI test are not affected by age. Nonetheless, females showed less decline compared to males at task failure, which is contrary to previous evidence showing similar VA changes between sexes after RI tests (Azevedo et al. 2023b). However, it must be highlighted that the former study had a 30s gap between exercise cessation and performance fatigability evaluation, which could have resulted in an underestimated exercise-induced decline in VA (Froyd et al. 2013). Indeed, whereas in a previous study, there was no VA decline after the RI tests (Azevedo et al. 2023a), and our current results showed a ~ 8% decrease from baseline (Fig. 2B). Thus, these results highlight the relevance of the methods chosen to evaluate exercise-induced VA responses as its recovery time course is faster than IMVC and contractile function variables (Froyd et al. 2013). Nonetheless, it could be speculated that males, regardless of the age, might produce a greater metabolic disturbance because of greater anaerobic metabolism reliance, which could indirectly affect the VA by exercise-induced muscle acidosis, probably acting on group III/IV muscle afferents in the interstitial space (Hureau et al. 2022).

### Characteristics of the study sample and its effects on overall results

The characteristic of our sample is another factor that must be considered when interpreting the current results since the aging process might affect individuals differently. For example, aging is accompanied by a decrease in muscle quality (i.e., force per kg of lean body mass) (Sundberg et al. 2018a), probably due to loss of type II fibers, motor units’ number and atrophy of the remaining (Hunter et al. 2016). It is noteworthy that the loss of maximal isometric force across the lifespan is more pronounced in males compared to females, probably because males have a greater loss of muscle mass and maximal force in the lower limbs, specially beyond ~ 60 years old (Lindle et al. 1997). However, our results showed that the sex differences in IMVC and contractile function variables at baseline were maintained regardless the aging process (i.e., older females vs males, and younger females vs males; Table 2). In fact, sex differences in body composition and muscle quality in our sample were maintained regardless of age (i.e., differences in total lean body mass and IMVC were similar between sexes regardless the age group at baseline), which might have diminished any possibility of identifying age and sex interaction effects in our results. Additionally, the aging process has been associated with lower VA at baseline, probably due to impairments in the motor cortex, spinal cord, motoneuron and neuromuscular junction (Hunter et al. 2016). However, it is uncertain if lower VA and associated age-related changes in underpinning mechanisms are related to aging process per se or other factors as fitness level (Harridge et al. 1999) and health issues (Rozand et al. 2020). For example, previous data have shown that some physiological parameters in active older individuals and master athletes are characterized as good as those observed in their sedentary and/or trained young counterparts (Gries et al. 2018; George et al. 2018). Thus, given that the older individuals in the current study displayed a fitness level (i.e., V̇O_2_max) above of the expected range for their age group (Gries et al. 2018), and that they were as physically active as the young group (i.e., see *participants section* for details). Therefore, it could be speculated that the age-related impairments in central nervous system function and muscle quality might have been minimized in our sample as the older participants were characterized with superior aerobic capacity when compared to the overall matched age population (Gries et al. 2018).

## Conclusion

The present study is the first to demonstrate that, at least in physically active individuals, age and sex effects in performance fatigability did not show an interaction, thus suggesting that sex-related differences in fatigability after a cycling RI test are preserved as we age. Moreover, whereas the maximal voluntary force is affected by both age and sex, the decline in contractile function is lower in older compared to younger individuals, and voluntary activation is more affected in males compared to females.

## Data Availability

All data supporting the findings of this study are available within the paper. Further data are available from the corresponding author on reasonable request.
